# Behavior of human periodontal ligament cells on dentin surfaces ablated with an ultra-short pulsed laser

**DOI:** 10.1038/s41598-017-12871-w

**Published:** 2017-10-06

**Authors:** Jing Liu, Oleh Andrukhov, Markus Laky, Sylvia Nürnberger, Andreas Moritz, Peijun Lyu, Xiaohui Rausch-Fan

**Affiliations:** 1 Center of Digital Dentistry, Peking University School and Hospital of Stomatology, Beijing, 100081 China; 2 Division of Conservative Dentistry and Periodontology, School of Dentistry, Medical University of Vienna, Vienna, Austria; 3 Department of Prosthodontics, Peking University School and Hospital of Stomatology, Beijing, 100081 China; 4 National Engineering Laboratory for Digital and Material Technology of Stomatology, Beijing, 100081 China; 5 Research Center of Engineering and Technology for Digital Dentistry, Ministry of Health, Beijing, 100081 China; 6 Ludwig Boltzmann Institute for Experimental and Clinical Traumatology, Austrian Cluster for Tissue Regeneration, AUVA Research Center, Vienna, Austria

## Abstract

This study aimed to evaluate the effects of an ultrashort pulsed laser (USPL) (1064 nm, 20 ps, 100 kHz) with different laser fluences (F, 4, 6, 8 J/cm^2^) and pulse overlaps (PO, 0, 50%) on human periodontal ligament cells (hPDLs) behavior. Dentin samples were ablated with USPL with different combinations of fluences and pulse overlaps; some samples were ablated with an Er:YAG laser (2940 nm, 150 µs, 100 mJ/pulse, 5 J/cm^2^) and some samples were ground with a carbide bur. Then hPDLs were grown on the samples after different treatments. Dentin morphology and cell adhesion were observed with SEM and gene expressions were measured by RT-PCR. The results showed dentin surfaces ablated with USPL when F = 4 J/cm^2^, PO = 0, and F = 6 J/cm^2^, PO = 0 were partially intact with obvious ridges and valleys and cells on these surfaces grew mostly along the valleys. USPL ablated surfaces in other groups were entirely ablated and cell cluster formation was observed. The RT-PCR results showed an upregulation of osteocalcin of cells grown on the dentin after some laser treatment. It can be concluded that USPL could improve the attachment and differentiation of hPDLs and thus potentially promote periodontal tissue regeneration.

## Introduction

Apicoectomy is an endodontic surgical procedure which is usually performed when a conventional root canal therapy has failed and a retreatment was already unsuccessful or is not advised^1^. The most traditional way to remove the root-end in clinic is the use of burs, and the root surface after different types of burs may have various patterns on the resected root surface^2^. The most important goal of apicoectomy is to produce a resected root-end with optimal conditions for subsequent regeneration of the periodontal ligament (PDL) across the resected root-end^3^. Different root surfaces may have different effects on the cell behavior like attachment and orientation^4^. Previous studies have shown that cell behavior is significantly influenced by the surface properties, including surface roughness and morphology^5,6^. After apicoectomy, cells in adjacent tissue become activated; they proliferate, migrate to the wounded area and produce growth factors and new matrix components^3^, which lead to bone generation, cementum deposition and PDL tissue formation^7^.

In last years, lasers were widely used for different dental treatments^8–18^, including root end resection^19,20^. This technique shows some advantages compared to traditional grinding tools like low level of discomfort and vibration^21^. Using the Er:YAG laser for apicoectomy has shown promising results^22^. In 1990s, the USPL drew the attention of researchers because its superior results for ablating dental hard tissues compared to both traditional grinding instruments and long-pulse laser systems^23^. The USPL removed tissue at lower energy levels, exhibited precise ablation depth and minimal thermal damages, low level of pain and reduced noise. These advantages owe to a special plasma-mediated ablation mechanism^24–26^. Previous studies show that ablating dental hard tissues using an USPL with appropriate parameters results in low levels of carbonization, melting, resolidification and microcracks, and the dentinal tubules remain mostly open. Moreover, the boundaries of the ablation cavities are clear and fine defined, the walls are steep and smooth, the non-irradiated areas exhibit no heat effects, and the original morphology is unchanged^25,27–29^.

Regarding the outstanding dental hard tissue ablation capability of USPL, it is interesting to apply this laser also in apicoectomy. However, the resected root surfaces after treatment with burs, Er:YAG laser and USPL may have different effects on cell behavior. Therefore, the present *in vitro* study investigated the behavior of primary human periodontal ligament cells on USPL ablated dentin surfaces.

## Results

### SEM observation of dentin surface and cell morphology

**Figure 1:**
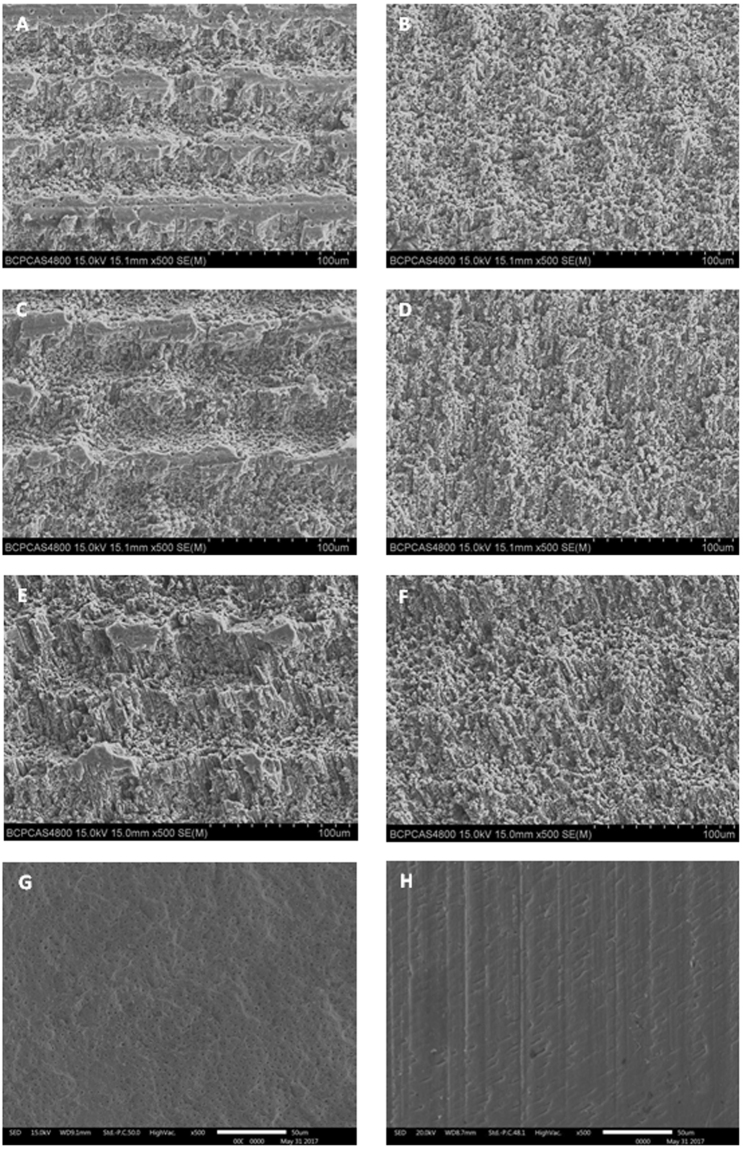
Representative of SEM images of dentin surface after treatment of each group. (**A**) ×500, G1 (F = 4 J/cm^2^, PO = 0), some of the dentin surface remained intact, clear ridges and valleys were produced, the dentin surface seemed rougher but regular; G2 (F = 4 J/cm^2^, PO = 50%), the dentin surfaces were flatter, no smear layer was observed; (**C**) ×500, G3 (F = 6 J/cm^2^, PO = 0), some of the dentin surface remained intact, clear ridges and valleys were produced, the dentin surface seemed rougher but regular; (**D**) ×500, G4 (F = 6 J/cm^2^, PO = 50%), the dentin surfaces were flatter, no smear layer was observed; (**E**) ×500, G5 (F = 8 J/cm^2^, PO = 0), the dentin surface were flatter, ridges and valleys were not obvious, no smear layer was observed; (**F**) ×500, G6 (F = 8 J/cm^2^, PO = 50%), the dentin surfaces were flatter, no smear layer was observed; G7 (Er:YAG laser), irregular and scaly morphology with open dentinal tubules could be observed, no smear layer could be observed; G8 (control group), smooth surface covered with debris, dentin tubules are closed based on smear layer.

The laser fluence and pulse overlap had remarkable effects on dentin surface structure after USPL ablation. Representative images of dentin after treatments are shown in Fig. 1. After ablation with F of 4 and 6 J/cm^2^ and PO = 0, in G1 (Fig. 1A) and G3 (Fig. 1C)), some of the dentin surface remained intact, clear ridges and valleys were produced, and the dentin surface seemed rougher but regular. When the highest F (8 J/cm^2^) and the higher PO (50%) were used, i e. G2 (Fig. 1B), G4 (Fig. 1D), G5 (Fig. 1E) and G6 (Fig. 1F)), the dentin surfaces were flatter than G1 and G3, no smear layer was observed. After ablation with Er:YAG laser (G7 (Fig. 1G)), image revealed irregular and scaly morphology with open dentinal tubules, no smear layer was observed. The control group treated with carbide bur (G8, control) displayed a smooth surface, which was covered with debris and smear layer, no open dentinal tubule was observed (Fig. 1H).

**Figure 2:**
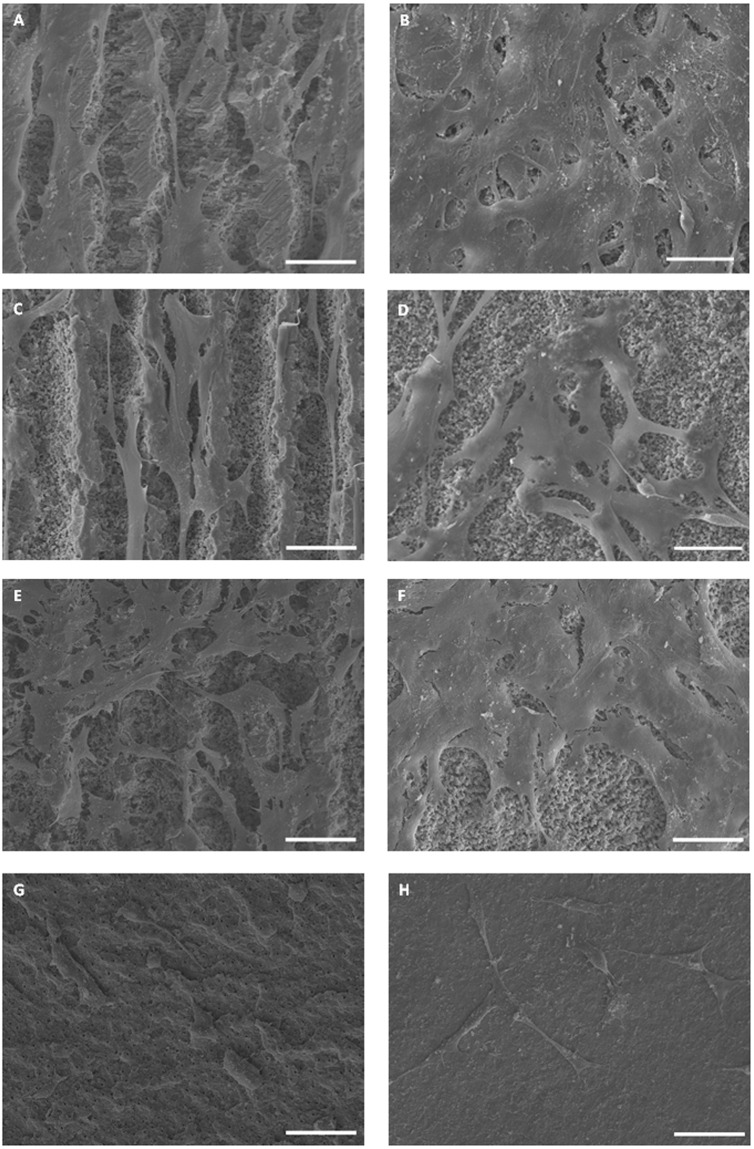
Representative SEM images of PDLs after 24 h incubation, horizontal bar in each image corresponds to 50 µm. (**A**) ×500, G1 (F = 4 J/cm^2^, PO = 0), cells spread and adhered mostly along the dentin valleys via long, expanded filopodia; (**B**) ×500, G2 (F = 4 J/cm^2^, PO = 50%), cells spread and adhered in all directions, polygonal cells merged with each other and formed cluster; (**C**) ×500, G3 (F = 6 J/cm^2^, PO = 0), cells spread and adhered mostly along the dentin valleys via long, expanded filopodia.; (**D**) ×500, G4 (F = 6 J/cm^2^, PO = 50%), cells spread and adhered in all directions, polygonal cells merged with each other and formed cluster; (**E**) ×500, G5 (F = 8 J/cm^2^, PO = 0), cells spread and adhered in all directions, polygonal cells merged with each other and formed cluster; (**F**) ×500, G6 (F = 8 J/cm^2^, PO = 50%), cells spread and adhered in all directions, polygonal cells merged with each other and formed cluster; (**G**) ×500, G7 (Er:YAG laser), some of the cells had a round shape with few short filopodia adhering to the material surface; (**H**) ×500, G8 (control group), dentin tubules are closed based on smear layer, cells adhered to the surface via thin filopodia, less cells were observed on the dentin surface.

Representative images of attached cells on different dentin surfaces after 24 h incubation time are shown in Fig. 2. After 24 h, in the USPL groups, the cells were flattened and attached to the dentin. In G1 (Fig. 2A) and G3 (Fig. 2C), cells spread and adhered mostly along the dentin valleys via long, expanded filopodia. In G2 (Fig. 2B), G4 (Fig. 2D), G5 (Fig. 2E) and G6 (Fig. 2F), cells were denser than in G1 and G3, spread and adhered in all directions, polygonal cells merged with each other and formed cluster. On the surface in G7 (Er:YAG laser group), there were only few cells attached with poorly developed process. Some of the cells had a round shape with few short filopodia adhering to the material surface (Fig. 2G). In G8 (control group), cells adhered to the surface via thin filopodia, less cells were observed on the dentin surface (Fig. 2H).

### Quantitative real-time PCR

**Figure 3:**
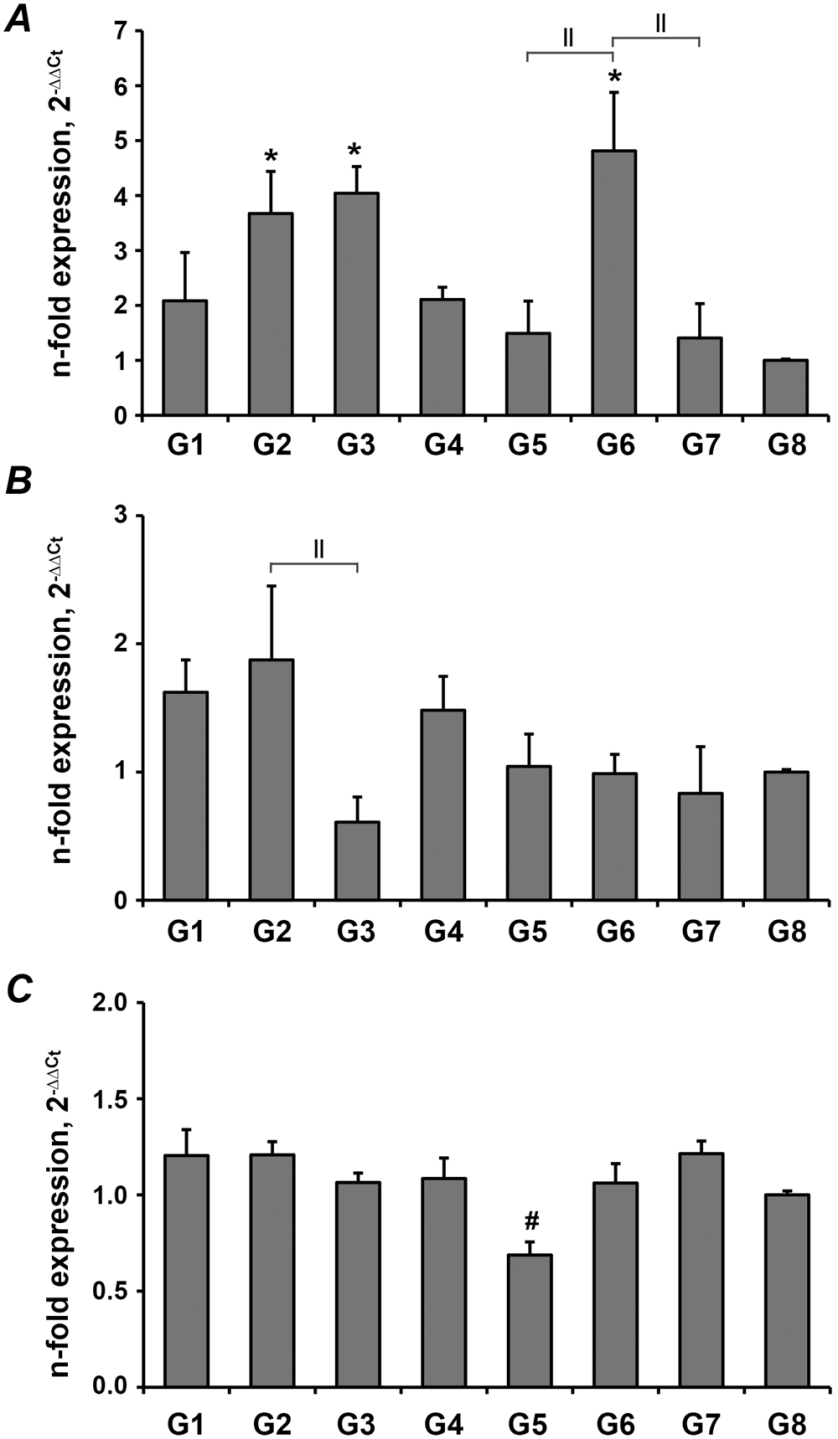
Gene expression of osteogenesis-related genes in PDLs cultured on the dental slices in normal growth media. Primary PDLs were cultured on dental slices with different laser treatment in DMEM media for 5 days and the expression of osteocalcin (**A**), alkaline phosphatase (**B**), and RUNX2 (**C**) was quantified by qPCR. Y-axes represent n-fold expression levels in relation to that in cells grown on the tissue culture plastic (G8)). *Significantly higher compared to control group, p < 0.05. ^#^Significantly lower compared to control group, p < 0.05. ^||^Significantly different between groups with different dentin slices treatment.

The expressions of osteogenic genes in the DMEM cultured groups are shown in Fig. 3, cells grown on G6 showed the highest value of OC. Bonferroni test revealed that the values of OC mRNA in G2, G3 and G6 are significantly increased compared to the control group (p = 0.009, 0.002 and 0.001 respectively), G6 was also significantly higher than G5 and G7 (Fig. 3A). There was no significant difference of the ALP mRNA level between laser-ablated groups and the control group (p>0.05), a significant difference existed only between G2 and G3 (p = 0.019) (Fig. 3B). The value of RUNX2 mRNA in G5 was significantly lower compared to the control group (p = 0.023, Fig. 3C).

**Figure 4:**
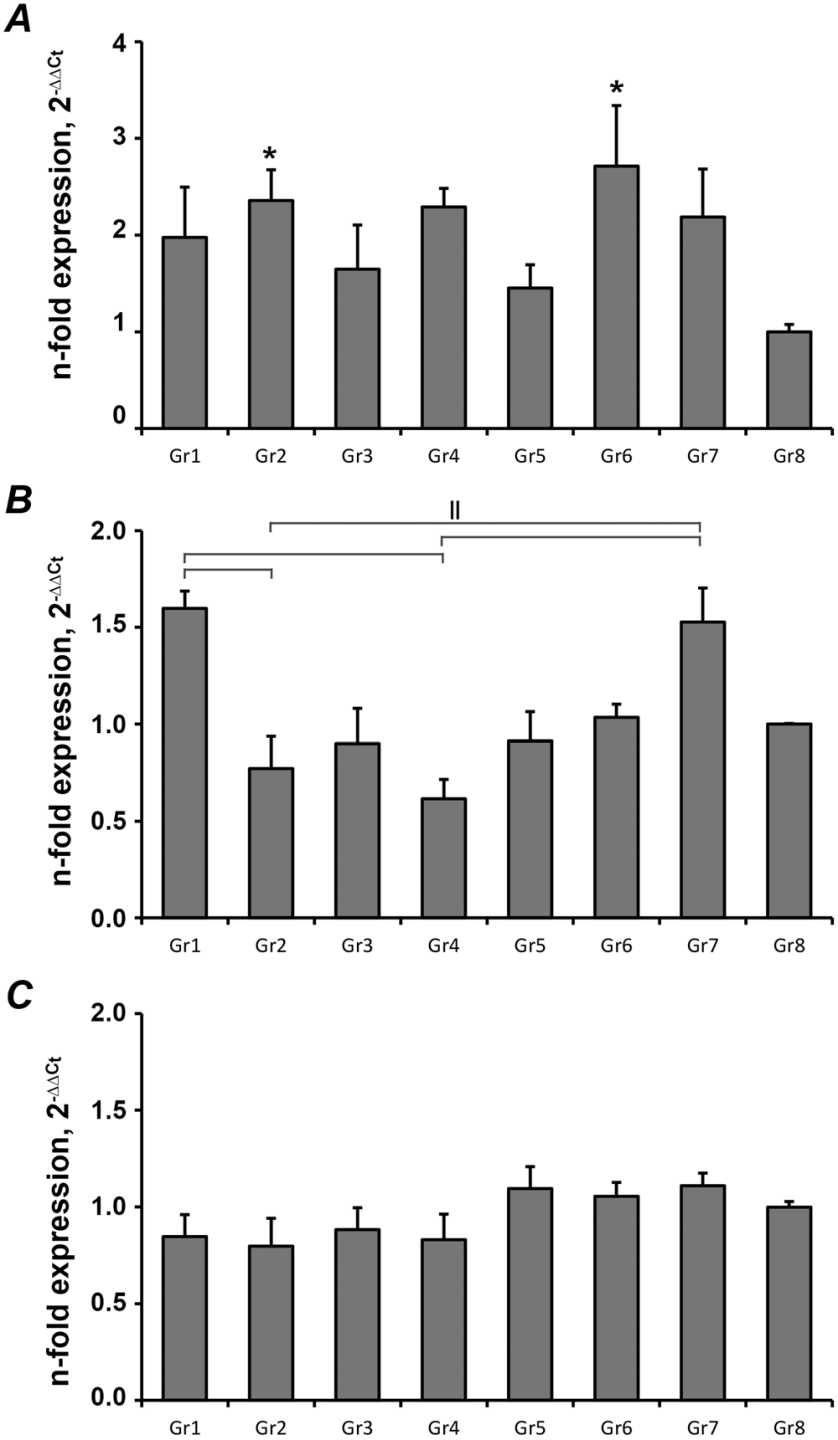
Gene expression of osteogenesis-related genes in PDLs cultured on the dental slices in osteogenic media. Primary PDLs were cultured on dental slices with different laser treatments in osteogenic media for 5 days and the expression of osteocalcin (**A**), alkaline phosphatase (**B**), and RUNX2 (**C**) was quantified by qPCR. Y-axes represent n-fold expression levels in relation to that in cells grown on the tissue culture plastic (G8)). ^*^Significantly higher compared to control group, p < 0.05. ^||^Significantly different between groups with different dentin slices treatment.

The expressions of osteogenic genes in the osteogenic medium cultured groups are shown in Fig. 4. The values of OC mRNA in G2 and G6 were significantly up-regulated compared to control group (p = 0.034 and 0.004 respectively), and G6 showed the highest OC mRNA level (Fig. 4A). There was no significant difference in the ALP mRNA expression between laser ablated groups and the control group (p>0.05). However, ALP mRNA value in G1 was significantly different from G2 and G4 (p = 0.004 and 0.000 respectively), G2 and G4 were also significantly different from G7 (p = 0.010 and 0.000 respectively) (Fig. 4B). There was no significant difference in the RUNX2 expression between all groups (p > 0.05, Fig. 4C).

## Discussion

The attachment of periodontal ligament cells to the resected root-end is one of the most important problems after apicoectomy^30^. PDLs may differentiate to different kinds of cells, including cementoblasts and osteoblasts, and therefore are essential for periodontal regeneration.

Conventionally, apicoectomy is performed with various kinds of burs, leading to smoother surface with smear layer and an increased risk for the presence of microorganisms and permeability^19^. The USPL has become the research hotspot in dentistry; however, this kind of laser has not been tried for apicoectomy, and related information is also rather rare. In the present study, USPL was used with different laser fluences and pulse overlaps because these two parameters are key factors that influence ablation outcome^31–33^. The USPL parameters setting in the present study referred to the previous study, in which morphology and wettability changes were found. Laser fluences of 4 J/cm^2^ to 8 J/cm^2^ were chosen because at lower laser fluence, no significant effect on the surface could be observed, whereas higher laser fluence results in melting and resolidification^34^. Each sample in G1-G6 was ablated 10 times to make sure the whole surface could be irradiated and the surface differences were obvious enough to be observed. The surfaces treated with USPL were compared with surfaces treated with an Er:YAG laser, which is commonly used for dentin preparation.

Compared to the bur ground dentin surface, the USPL ablated surfaces were rougher and without any smear layer. It can be seen that especially in G1 and G3 surfaces, which were treated with lower laser fluence and lower pulse overlap, dentin surfaces were rougher with obvious ridges and valleys. The surfaces seemed to be flatter when laser were used with higher fluence or pulse overlap. These findings can be explained by a fact that surface is ablated with more power at a higher laser fluence, which results in removing thicker surface layer and make it flatter and less rough. At PO of 50%, each focal point is effectively ablated twice, which in this case appeared to significantly reduce the influence of laser fluence on surface roughness, at least at the levels tested here. The influences of repetition time and different setting of PO within one scanning line and between two scanning lines were not studied here. The results described in this manuscript were concluded according to the method above. The influences and mechanism of repetition time and PO will be taken as potential research topics in future studies.

The Er:YAG laser irradiated dentin surface was rough and typically scaly. Previous studies have shown that the surface roughness of Er:YAG laser irradiated root surface does not depend on the angulation of the working tip or the output power^35^. However, with increasing output power, carbonization and melting could occur^36^, which could hinder the attachment of hPDLs^37^.

In the present study, the morphological roughness of the lased dentin surface seemed to have effects on attachment and differentiation of hPDLs. Cell morphology contact area of cells can be regarded as an indicator for the affinity of the cells. Flat cells usually are firmly attached to the surface by their extended cell body and lamellipodia, while round cells can be considered to be poorly attached^38^. In this study, according to SEM images, well-attached hPDLs were observed in all USPL ablated dentin surfaces, while cells on Er:YAG laser irradiated and bur ground dentin surfaces were rounder and less attached (Fig. 2), which was different from previous studies^30,39^. The SEM images also showed hPDLs attachment and cluster formation on dentin surfaces G2, G4, G5 and G6. The differences in the cell attachment seemed to correlate with surface structure, as G2, G4, G5, G6 had flatter surface characteristics than G1 and G3.

Isotropy and anisotropy of a surface could be one factor that influences cell behavior on this surface. On the one hand, an isotropic topography is shown to have no influence on the cell morphology, but it highly induces cell motility. On the other hand, cells cultured on anisotropic topographies are highly elongated and aligned, and the lowest motility was observed on a ridge and valley ratio of 1:1 and 1:3.^40^. In this study, surfaces in G1, G3 could be defined as anisotropic surfaces because of obvious ridges and valleys. Due to the ablation method, the ratio of ridge and valley in G1 and G3 were almost 1:1. This may explain the morphology of hPDLs in G1 and G3. The isotropy of G2, G4, G5 and G6 could also accelerate cluster formation by inducing cells motility.

The expressions of three markers of the osteogenic differentiation were investigated. OC is implicated in bone mineralization and calcium ion homeostasis^41^, also plays an important role in bone mineralization^42^. As OC is produced by osteoblasts, it is often used as a marker for the bone formation process, and an increasing expression of OC could indicate the stimulation of osteogenesis. ALP is the most important enzyme for bone matrix mineralization in initial stages of osteoblastic differentiation^43^. RUNX2 has been proven as an essential transcriptional factor for the differentiation of osteoblasts, and the increased expression of RUNX2 can lead to upregulation of osteogenic differentiation-related genes^44^.

In this study, coronal dentin instead of root dentin was used because it is easier to get the standardized samples from different individuals. The root dentin is more heterogeneous between different individuals and this might have an influence on the data. Root dentin is indeed different from the coronal dentin in some physiological aspects, but the main component and constitution are same, which are the most important factors that affect the ablation outcome of laser ablation. This is a potential limitation of the present study, as primary result has been concluded; further experiments will be conducted with root dentin which is more close to clinic.

Another possible limitation of this study is the possible effect of temperature on the dentin surface structure suggested by previous study^45^. Previous study suggests that under conditions used in our experiments surface ablation occurs largely due to non-thermal mechanism^46^. A potential effect of the temperature on dentin surface could be further minimized by optimization of scanning speed. Exact contribution of the thermal component in USPL dentin surface ablation must be investigated by further appropriately designed studies.

## Conclusion

USPL can improve the attachment of hPDLs compared with Er:YAG laser and traditional rotary instrument. In G2, F = 4 J/cm^2^, PO = 50%; G4, F = 6 J/cm^2^, PO = 50%; G5, F = 8 J/cm^2^, PO = 0; G6, F = 8 J/cm^2^, PO = 50%, more hPDLs were attached and formed cluster. The expression of OC in G2 and G6 is up-regulated in both normal medium and osteogenic medium, which means the ultra-short pulsed laser together with appropriate parameters, could improve the differentiation of hPDLs.

## Methods

### Specimen preparation

Thirty-two intact, caries-free human third molars were used in this study. Informed consent was obtained from all subjects, experimental protocols were approved by the ethics committee of University Clinic of Dentistry, Medical University of Vienna, and the methods were carried out in accordance with the relevant guidelines and regulations. After extraction, the teeth were cleaned and soaked in Hanks buffer. The teeth were cut into crowns and roots along the cementum-enamel junction using Exakt cutting instrument (Exakt Apparatebau, Norderstedt, Germany), then the crowns were cut longitudinally into slices of approximately 1.5mm in thickness. 96 slices produced from 32 different teeth were used in the present study. After cutting the slices, the enamel was removed with bur.

### Samples treatment

The ultra-short pulsed Nd:YVO_4_ laser, developed by the Institute of Physics (Chinese Academy of Sciences, Beijing, China) was used in the present study. The emitted light has a wavelength of 1064 nm, pulse duration (τ) of 20 ps, pulse repetition rate (f) of 100 kHz, and the output power up to 20 W. A galvanometric scanning system (C610, Daheng Laser, Beijing, China) was used to focus the laser beam on sample surface. The spot diameter (φ) at the focal plane was about 40 μm. Groups from 1 to 6 were ablated using this laser. The dentin samples were fixed on a stage with dentin surfaces oriented at the focal plane. Parallel lines were irradiated onto samples with different laser fluence (F) and pulse overlap (PO). Fluence (J/cm^2^) = [Out power (W)·Pulse duration (s)]/Area (π(φ/2/)^2^)(cm^2^). The PO was determined by scanning speed (v), scanning line spacing (s), f and φ. The PO within one scanning line can be described as ((φ − v/f)/φ, and the PO between two scanning lines can be described as s∕φ. Teeth slices were irradiated with three different fluences: 4 J/cm^2^, 6 J/cm^2^, and 8 J/cm^2^. The out power for each fluence could be calculated according the formula: out power = F × π (φ/2)^2^ × f. PO of either 0 or 50% was applied for each fluence. Thus 6 groups of dentin slices with different surface treatment protocol were ablated with USPL: group 1 (G1), F = 4 J/cm^2^, PO = 0; G2, F = 4 J/cm^2^, PO = 50 %; G3, F = J/cm^2^, PO = 0; G4, F = 6 J/cm^2^, PO = 50 %; G5, F = 8 J/cm^2^, PO = 0; G6, F = 8 J/cm^2^, PO = 50%. Each sample in G1-G6 was ablated 10 times. Then samples irradiated with Nd:YVO_4_ were compared with those irradiated with Er:YAG laser (group 7, G7) (Laser: LiteTouch, Syneron Dental Lasers, Israel) with following parameters: wavelength of 2940 nm, output energy of 100 mJ/pulse, pulse repetition rate of 10 Hz, pulse duration of 150 µs, and fluence of 5 J/cm^2^. A straight tip (Syneron Dental Lasers, Israel) with diameter of 800 μm was used for samples irradiation. The tip was moved in non-contact mode approximately 1 mm from the surface in a sweeping manner to irradiate the entire dentin surface. The angle between tip and dentin surface was 45°, and the tip was moved away from the irradiation sites. The entire dentin surface was irradiated 4 to 5 times in order to resemble the clinical procedure as described by Crespi *et al*.^47^. All irradiations were performed by one operator. Non-irradiated samples were used as a control (group 8, G8). These samples were ground with a carbide bur under water irrigation.

### Cell culture

Primary human periodontal ligament cells were isolated as described in our previous studies^48^. hPDLs were isolated from healthy patients undergoing routine extraction of their third molar teeth. Patients were informed in details before the surgical procedures and gave their written agreement. The study protocol was approved by the Ethics Committee of the Medical University of Vienna. hPDLs were isolated by scraping the ligament tissue from the teeth root surface and cultured in Dulbecco’s modified Eagle’s medium (DMEM), supplemented with 10% fetal bovine serum (FBS), streptomycin (50 µg/ml) and penicillin (100 U/ml) under humidified air atmosphere of 5% CO_2_ at 37 °C.

### Scanning electron microscopy of the dentin surface and cell morphology

hPDLs were seeded in wells of 4-well-plate containing the dentin slices with different treatment at a density of 1 × 10^5^ cells/well and cultured for 24 h in DMEM. Two samples from each group were randomly chosen to evaluate the morphology of PDLs on USPL and Er:YAG laser ablated dentin surface. After 24 h culturing, the samples were rinsed with PBS to remove unattached cells. Then the samples were fixed with 4% formalin for 24 h and dehydrated in a grade series of ethanol and chemically dried with Hexamethyldisilazane (HMDS, Sigma-Aldrich). Finally, the samples were coated with gold and observed with a SEM (JEOL - JSM IT 300, Jeol, Japan) at an accelerating voltage of 15 kV.

### Quantitative real-time PCR

For gene expression analysis, samples were placed in 24-well plates; hPDLs were seeded and cultured in either standard DMEM medium or osteogenic medium for 5 days. Osteogenic medium was an alpha modified minimal essential medium (α-MEM) supplemented with 10 % FCS, 1 % penicillin/streptomycin, 0.1 µM dexamethasone (Sigma, St. Louis, USA), 10 mM β-glycerophosphate (Alfa Aesar, Karlsruhe, Germany), and 50 µM L-ascorbic acid-2-phosphate (Sigma, St. Louis, USA). Three different samples for each laser treatment were used in each experiment. The cells were first seeded in 30 µl medium, so that only the dentin slices were covered with medium. Cells were allowed to attach for 4 h and afterward 500 µl medium was added into each well. After 5 days culture, cells were collected and the expression of osteogenesis related genes was analyzed by qPCR similarly to the methods used in our previous studies^49,50^ taking β2-microglobulin (β2M) as a house-keeping gene. Isolation of cellular mRNA, its transcription into cDNA, and quantitative analysis of specific DNA was performed using the TaqMan Gene Expression Cells-to-CT kit (Ambion/Applied Biosystems, Foster City, CA, USA) according to the manufacturer's instructions. qPCR was performed on ABI StepOnePlus device (Applied Biosystems) using the Taqman gene expression assays (Applied Biosystems) with following ID numbers: β2M, Hs99999907_m1; osteocalcin (OC), Hs00609452_g1; alkaline phosphatase (ALP), Hs01029144_m1; runt-related transcription factor 2 (RUNX2), Hs00231692_m1. qPCR reactions were performed in triplicate in 96-well plates using the following thermocycling conditions: 95 °C for 10 minutes, 50 cycles each for 15 s at 95 °C and 1 min at 60 °C. The point at which the PCR product was first detected above a fixed threshold (termed cycle threshold, C_t_) was determined for each sample. Changes in the expression of target gene were calculated using 2^−ΔΔCt^, where ΔΔC_t_ = (C_t_^target^ − C_t_^β2M^)_sample_ − (C_t_^target^ − C_t_^β2M^)_control_. The gene expression analyses were performed with cells mRNA from four different donors, and each gene was tested in triplicate. Cells growing on the bur ground dentin slices, which were not subjected to the laser irradiation, were used as a control. In each experiment, 3 different slices were used for each group.

### Statistical analysis

The normal distribution of all data was tested with Kolmogorov-Smirnov test. After confirming normal distribution, the statistical differences between different groups were analyzed by one-way analysis of variance (ANOVA) followed by Bonferroni post-hoc test for pairwise comparison. All statistical analysis was performed using statistical program SPSS 21.0 (SPSS, Chicago, IL, USA). Data are expressed as mean ± S.E.M. Differences were considered to be statistically significant at p < 0.05.

### Data Availability

All data generated or analyzed during this study are included in this published article.

## References

[1] Salgado RJC (2009). Comparison of different irrigants on calcium hydroxide medication removal: microscopic cleanliness evaluation. Oral Surgery, Oral Medicine, Oral Pathology, Oral Radiology, and Endodontology.

[2] Camargo Villela Berbert FL (2010). An *in vitro* evaluation of apicoectomies and retropreparations using different methods. Oral Surgery, Oral Medicine, Oral Pathology, Oral Radiology, and Endodontology.

[3] Al-Nazhan S (2004). SEM observations of the attachment of human periodontal ligament fibroblasts to non-demineralized dentin surface *in vitro*. Oral Surgery, Oral Medicine, Oral Pathology, Oral Radiology, and Endodontology.

[4] Bolortuya G (2011). Initial fibroblast attachment to Erbium:YAG laser-irradiated dentine. INT ENDOD J.

[5] Wirth C (2008). Biomaterial surface properties modulate *in vitro* rat calvaria osteoblasts response: Roughness and or chemistry. Materials Science and Engineering: C.

[6] Nakamura, M. *et al*. Polarized hydroxyapatite promotes spread and motility of osteoblastic cells. J BIOMED MATER RES A 9999A NA-NA (2009). 10.1002/jbm.a.32404 19274714

[7] Balto H, Al-Nazhan S (2003). Attachment of human periodontal ligament fibroblasts to 3 different root-end filling materials: Scanning electron microscope observation. Oral Surgery, Oral Medicine, Oral Pathology, Oral Radiology, and Endodontology.

[8] Keller U, Hibst R (1989). Experimental studies of the application of the Er:YAG laser on dental hard substances: II. Light microscopic and SEM investigations. LASER SURG MED.

[9] Hibst R, Keller U (1989). Experimental studies of the application of the Er:YAG laser on dental hard substances: I. Measurement of the ablation rate. Lasers Surg Med.

[10] Le Goff A, Dautel-Morazin A, Guigand M, Vulcain JM, Bonnaure-Mallet M (1999). An evaluation of the CO2 laser for endodontic disinfection. J Endod.

[11] Altshuler GB, Belikov AV, Sinelnik YA (2001). A laser-abrasive method for the cutting of enamel and dentin. LASER SURG MED.

[12] Liu J, Lai Y, Shu W, Lee S (2006). Acceptance and efficiency of Er:YAG laser for cavity preparation in children. Photomedicine and Laser Therapy.

[13] Ekworapoj P, Sidhu SK, McCabe JF (2007). Effect of different power parameters of Er, Cr: YSGG laser on human dentine. LASER MED SCI.

[14] Kim SS, Park JI, Lee JI, Kim GS, Cho HW (2008). Effects of laser-irradiated dentin on shear bond strength of composite resin. The Journal of Korean Academy of Prosthodontics.

[15] Youssef M (2008). Dentinal surface-cutting efficiency using a high-speed diamond bur, ultrasound and laser. LASER PHYS.

[16] Almehdi A (2013). Histological and SEM analysis of root cementum following irradiation with Er:YAG and CO_2_ lasers. Lasers Med Sci.

[17] Shahabi, S., Chiniforush, N. & Juybanpoor, N., Morphological Changes of Human Dentin after Erbium-Doped Yttrium Aluminum Garnet (Er:YAG) and Carbon Dioxide (CO_2_) Laser Irradiation and Acid-etch Technique: An Scanning Electron Microscopic (SEM) Evaluation. Journal of Lasers in Medical Sciences 4 (2013). PMC4281973 25606306

[18] Dostalova T, Jelinkova H (2013). Lasers in dentistry: overview and perspectives. PHOTOMED LASER SURG.

[19] Gouw-Soares S (2004). Comparative study of dentine permeability after apicectomy and surface treatment with 9.6 microm TEA CO_2_ and Er:YAG laser irradiation. J Clin Laser Med Surg.

[20] Arisu HD, Sadik B, Bala O, Türköz E (2008). Computer-assisted evaluation of microleakage after apical resection with laser and conventional techniques. LASER MED SCI.

[21] Leco BM, Martinez GJ, Donado RM (2007). Clinical and radiological course in apicoectomies with the Erbium:YAG laser. Med Oral Patol Oral Cir Bucal.

[22] Israel M, Cobb CM, Rossmann JA, Spencer P (1997). The effects of CO2, Nd:YAG and Er:YAG lasers with and without surface coolant on tooth root surfaces. An *in vitro* study. J CLIN PERIODONTOL.

[23] Momma C, Nolte SN, Chichkov B, T U Nnermann A, Others (1997). Precise laser ablation with ultrashort pulses. APPL SURF SCI.

[24] Gamaly EG, Rode AV, Luther-Davies B, Tikhonchuk VT (2002). Ablation of solids by femtosecond lasers: Ablation mechanism and ablation thresholds for metals and dielectrics. PHYS PLASMAS.

[25] Niemz MH (2004). Tooth ablation using a CPA-free thin disk femtosecond laser system. Applied Physics B: Lasers and Optics.

[26] Rubenchik AM, Feit MD, Perry MD, Larsen JT (1998). Numerical simulation of ultra-short laser pulse energy deposition and bulk transport for material processing. APPL SURF SCI.

[27] Luengo MC (2013). Evaluation of micromorphological changes in tooth enamel after mechanical and ultrafast laser preparation of surface cavities. Lasers Med Sci.

[28] Ji L (2012). Ti:sapphire femtosecond laser ablation of dental enamel, dentine, and cementum. Lasers Med Sci.

[29] Portillo MM (2012). Morphological alterations in dentine after mechanical treatment and ultrashort pulse laser irradiation. Lasers Med Sci.

[30] Bolortuya G (2012). Effects of dentin surface modifications treated with Er:YAG and Nd:YAG laser irradiation on fibroblast cell adhesion. Photomed Laser Surg.

[31] Lorenzo, M. C. *et al*. Ultrashort pulsed laser conditioning of human enamel: *in vitro* study of the influence of geometrical processing parameters on shear bond strength of orthodontic brackets. Lasers Med Sci 1 (2013). 10.1007/s10103-013-1491-2 24249356

[32] Chen H (2013). Effects of fluence and scanning velocity on the ablation efficiency of dentin and enamel by femtosecond laser. Zhonghua kou qiang yi xue za zhi = Zhonghua kouqiang yixue zazhi = Chinese journal of stomatology.

[33] Schelle, F. *et al*. Ultrashort pulsed laser (USPL) application in dentistry: basic investigations of ablation rates and thresholds on oral hard tissue and restorative materials. *LASER MED SCI* **1** (2013). 10.1007/s10103-013-1315-4 23609558

[34] Liu J (2015). Surface roughness and wettability of dentin ablated with ultrashort pulsed laser. J Biomed Opt.

[35] Folwaczny M, George G, Thiele L, Mehl A, Hickel R (2002). Root surface roughness following Er:YAG laser irradiation at different radiation energies and working tip angulations. J CLIN PERIODONTOL.

[36] Cobb CM (2006). Lasers in Periodontics: A Review of the Literature. J PERIODONTOL.

[37] Fayad MI, Hawkinson R, Daniel J, Hao J (2004). The effect of CO_2_ laser irradiation on PDL cell attachment to resected root surfaces. Oral Surgery, Oral Medicine, Oral Pathology, Oral Radiology, and Endodontology.

[38] Schwarz F (2003). *In vivo* effects of an Er:YAG Laser, an ultrasonic system and scaling and root planing on the biocompatibility of periodontally diseased root surfaces in cultures of human PDL fibroblasts. LASER SURG MED.

[39] Feist IS (2003). Adhesion and growth of cultured human gingival fibroblasts on periodontally involved root surfaces treated by Er:YAG laser. J PERIODONTOL.

[40] Lamers E (2010). The influence of nanoscale topographical cues on initial osteoblast morphology and migration. Eur Cell Mater.

[41] Lee NK (2007). Endocrine Regulation of Energy Metabolism by the Skeleton. CELL.

[42] Lian JB, Stein GS (1992). Concepts of osteoblast growth and differentiation: basis for modulation of bone cell development and tissue formation. Crit Rev Oral Biol Med.

[43] Wakabayashi S (2002). Involvement of phosphodiesterase isozymes in osteoblastic differentiation. J BONE MINER RES.

[44] Taniguchi, Y., Kakura, K., Yamamoto, K., Kido, H. & Yamazaki, J. Accelerated Osteogenic Differentiation and Bone Formation on Zirconia with Surface Grooves Created with Fiber Laser Irradiation. *CLIN**IMPLANT DENT R n/a-n/a* (2015). 10.1111/cid.12366 26179832

[45] Schelle, F., Meister, J., Oehme, B. & Frentzen, M., Transmission of 1064 nm laser radiation during ablation with an ultra-short pulse laser (USPL) system. 8208 (2012)

[46] Chichkov BN, Momma C, Nolte S, Von Alvensleben F, Nnermann TU (1996). A., Femtosecond, picosecond and nanosecond laser ablation of solids. Applied Physics A: Materials Science \& Processing.

[47] Crespi R, Capparè P, Toscanelli I, Gherlone E, Romanos GE (2007). Effects of Er:YAG laser compared to ultrasonic scaler in periodontal treatment: a 2-year follow-up split-mouth clinical study. J PERIODONTOL.

[48] Andrukhov O (2014). Both 25-Hydroxyvitamin-D3 and 1,25-Dihydroxyvitamin-D3 Reduces Inflammatory Response in Human Periodontal Ligament Cells. PLOS ONE.

[49] Fleischmann L (2015). Behavior of osteoblasts on TI surface with two different coating designed for orthodontic devices. J Mater Sci Mater Med.

[50] Shi B, Andrukhov O, Berner S, Schedle A, Rausch-Fan X (2014). The angiogenic behaviors of human umbilical vein endothelial cells (HUVEC) in co-culture with osteoblast-like cells (MG-63) on different titanium surfaces. DENT MATER.

